# Supplementary material for: Activation/Inhibition of Cholinesterases by Excess Substrate: Interpretation of the Phenomenological b Factor in Steady-State Rate Equation
Source: Int J Mol Sci. 2023 Jun 21;24(13):10472. doi: 10.3390/ijms241310472 (PMC10341919; doi:10.3390/ijms241310472)

Supplementary Information

Activation/Inhibition of cholinesterases by excess substrate: interpretation of the phenomenological « *b* » factor in steady-state rate equation

Aliya R. Mukhametgalieva[1], Andrey V. Nemtarev[2], Viktor V. Sykaev[2],

Tatiana N. Pashirova[2], Patrick Masson[1*]

[1] Kazan Federal University, Biochemical Neuropharmacology Laboratory,

18 ul. Kremlevskaya, 48002 Kazan, Russian Federation

[2]Arbuzov Institute of Organic and Physical Chemistry, FRC Kazan Scientific Center,

Russian Academy of Sciences, 8 ul. Arbuzov, Kazan, 420088, Russian Federation

* Corresponding author

pym.masson@free.fr

Content

1. Synthesis of ATMA: ^{1}H- and ^{13}C-NMR spectra

Figure S1. ^{1}H NMR spectrum of ATMA

Figure S2. ^{13}C-{^{1}H} NMR spectrum of ATMA

2. Benzoylcholine chloride solutions

The Fourier transform pulsed-gradient spin-echo (FT-PGSE) experiments were performed by BPP-STE-LED (bipolar pulse pair–stimulated echo-longitudinal Eddy current delay) sequence as described in [D. Wu, A. Chen and C.S. Johnson, J. Magn. Reson. 115 (1995) 260.]
The diffusion experiments were performed at least three times. All separated peaks were analyzed and the average values for the species were presented. The temperature was set and controlled at 30^{0} C with a 640 l/h airflow rate in order to avoid any temperature fluctuations owing to sample heating during the magnetic field pulse gradients.

Figure S3. ^{1}H-NMR spectrum of BzCh in 0.1 M phosphate-D_2O pH = 8.0

Figure S4. The self-diffusion coefficient of BzCh vs concentration (A) and reciprocal concentration (B) at 30 °C, in 0.1 M phosphate-D_2O, pH = 8.0.

Figure S5. Surface tension isotherms of BzCh

Figure S6. Absorption profile of BzCh and Absorbance at 232

Figure S7. Particle size distribution of BzCh using the Intensity and Number parameters

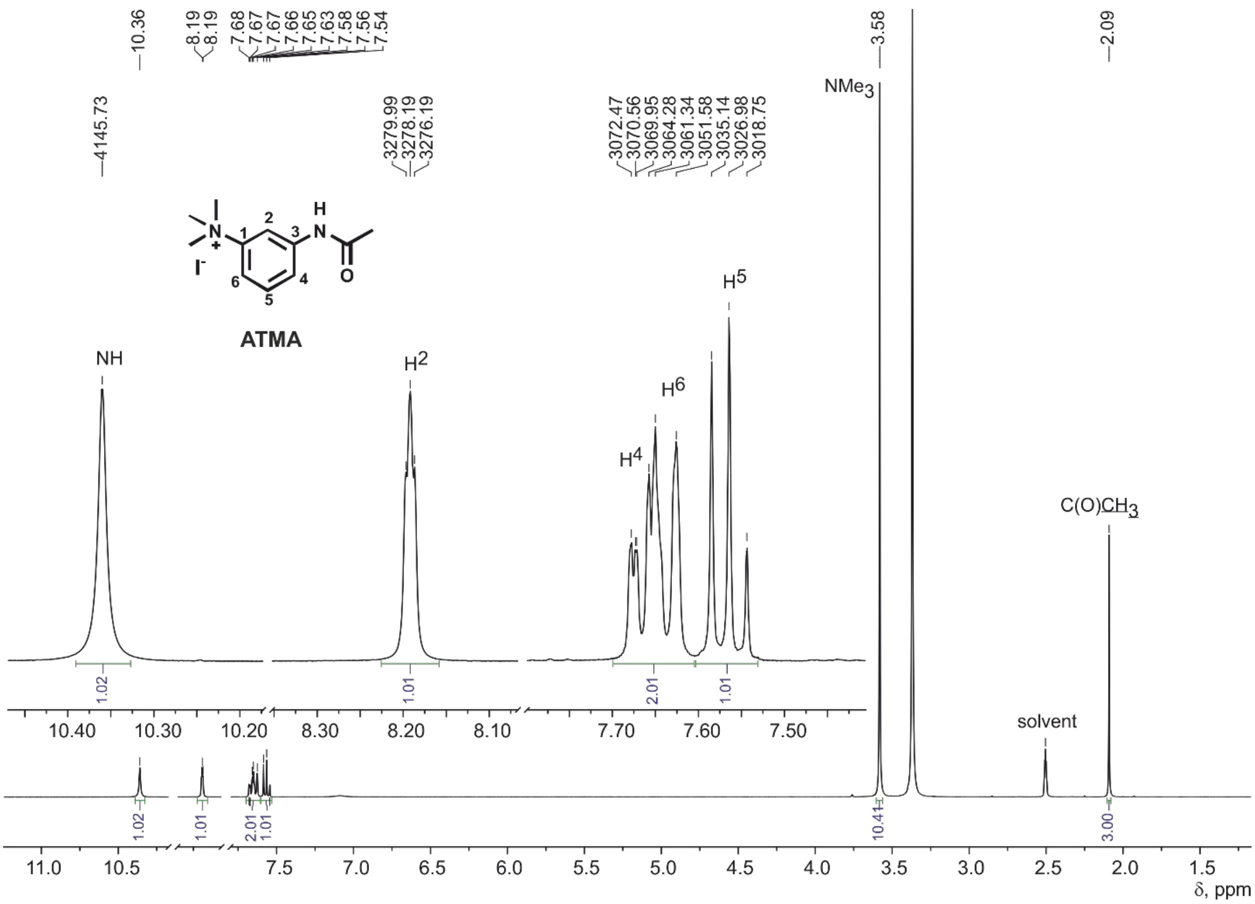

Figure S1. ^{1}H NMR spectrum of ATMA (400 MHz, DMSO-d_6)

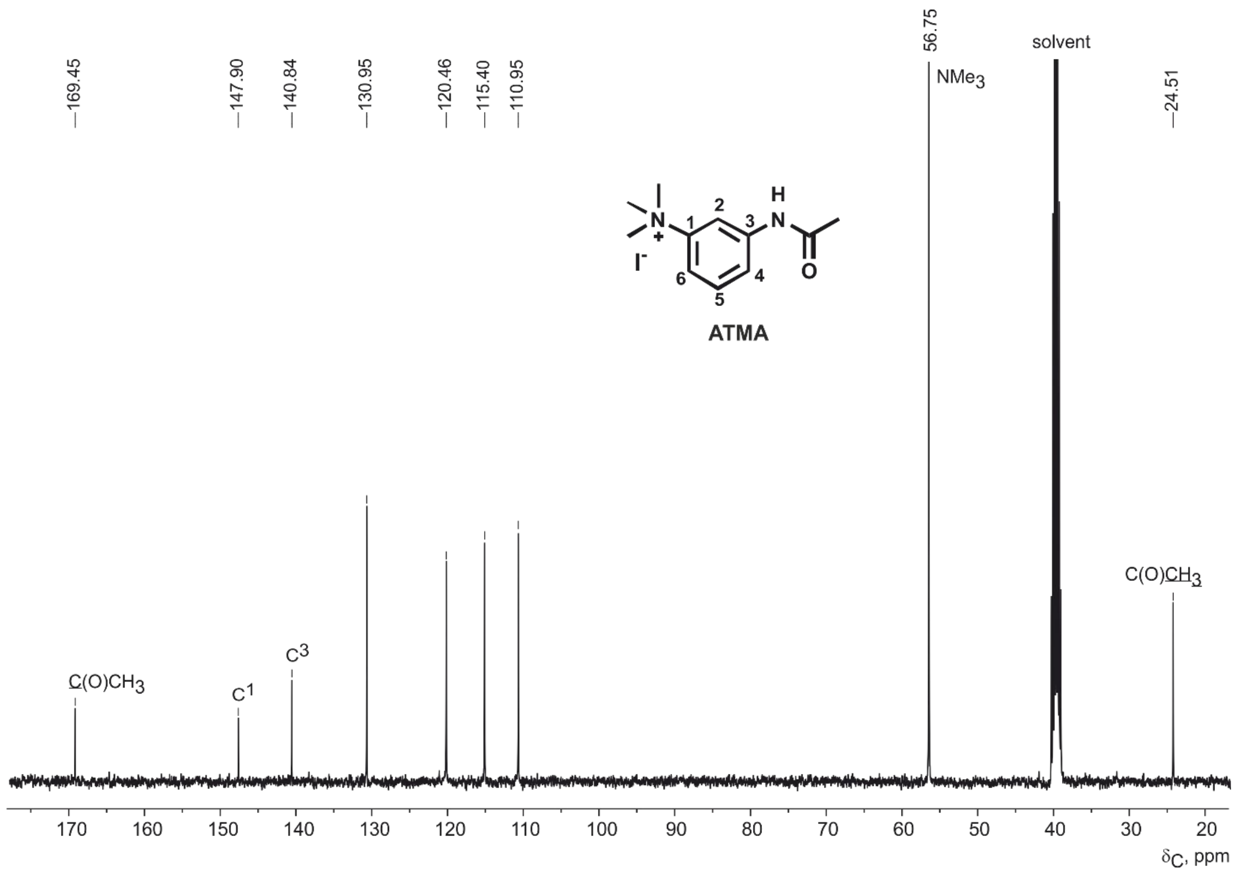

Figure S2. $^{13}C-\{^1H\}$ NMR spectrum of ATMA (100.6 MHz, DMSO-d$_6$)

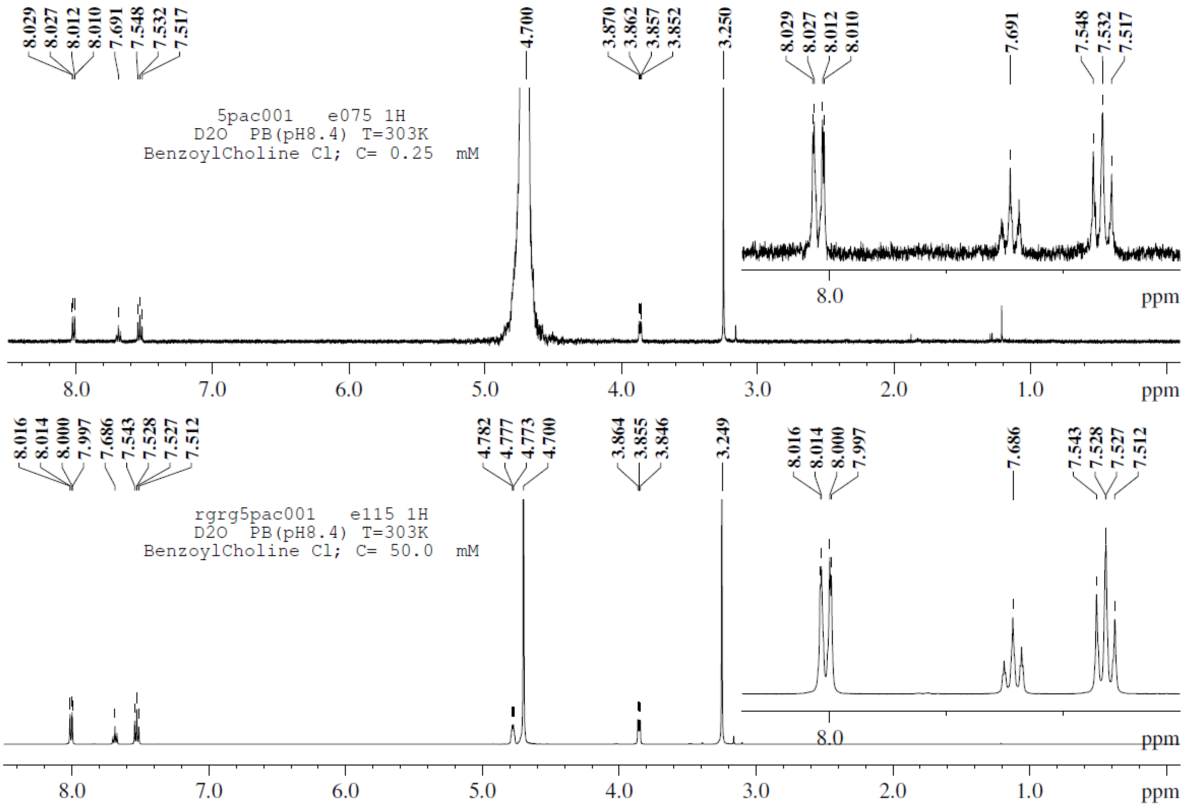

Figure S3.^{1}H-NMR spectrum of BzCh in 0.1 M phosphate-D_2O, pH = 8.0

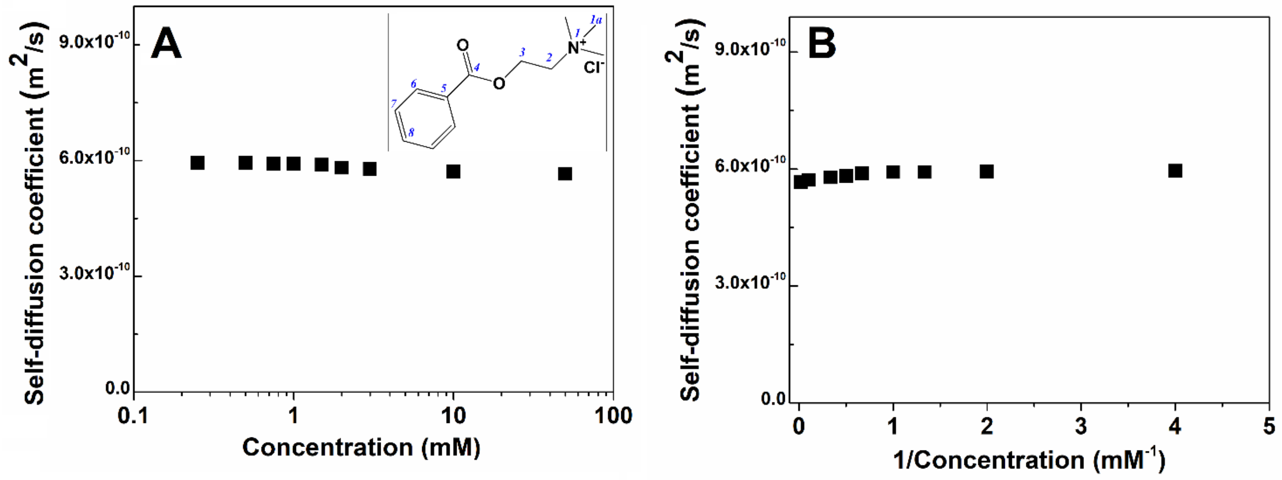

Figure S4. The self-diffusion coefficient of BzCh vs concentration (A) and reciprocal concentration (B) at 30 °C, in 0.1 M phosphate-D_2O, pH = 8.0.

Table S1. The self-diffusion coefficients (Ds), hydrodynamic radius (R_H) and aggregation numbers (Nagr) at various concentration of BzCh in 0.1 M phosphate-D_2O, 30°C

C, mM	0.25	0.5	0.75	1	1.5	2	3	10	50
Ds, m^2/s	5.95	5.94	5.92	5.92	5.89	5.82	5.79	5.72	5.67
Rh, Å	4.7	4.7	4.7	4.7	4.7	4.8	4.8	4.9	4.9
Nagr	1.0	1.0	1.0	1.0	1.0	1.1	1.1	1.1	1.2

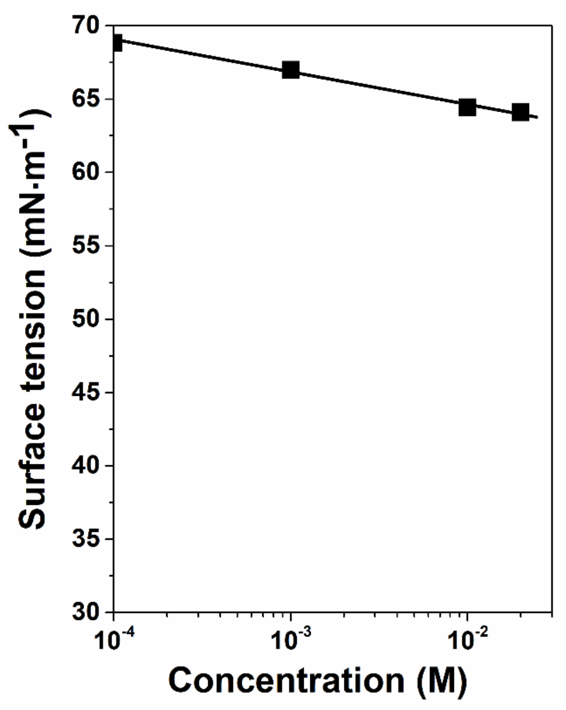

Figure S5. Surface tension isotherms of BzCh in 0.1 M phosphate buffer, pH = 8.0, 25 °C.

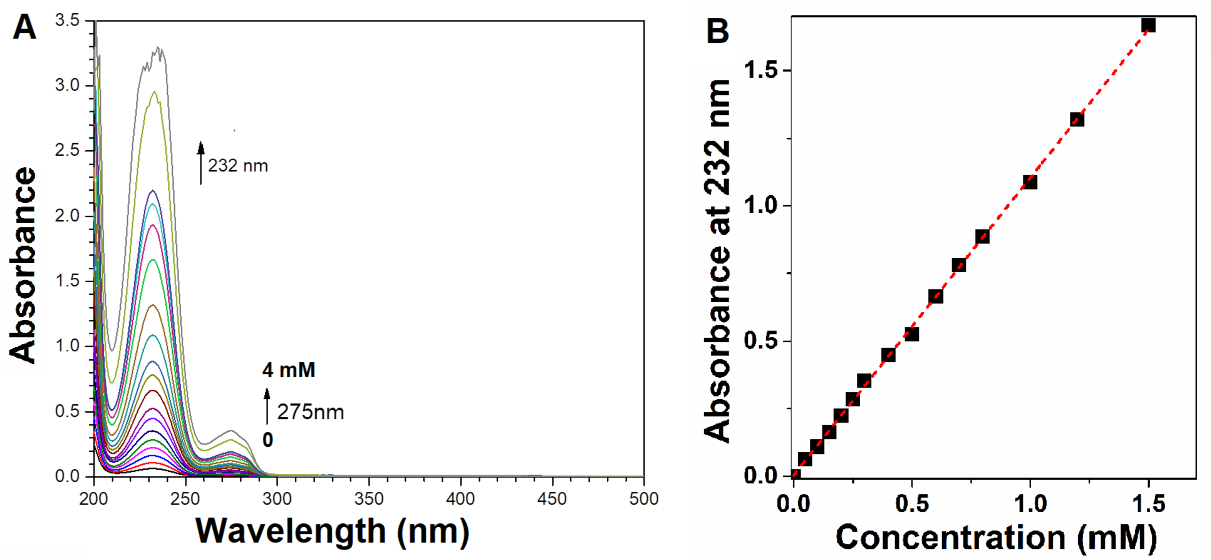

Figure S6. Absorption profile of BzCh (A) and Absorbance at 232 nm with increasing of concentration of BzCh (B), BzCh in 0.1 M phosphate pH = 8.0, L = 0.1cm, 25 ºC, C_{BzCh} (mM) = 0.05, 0.1, 0.15, 0.2, 0.25, 0.3, 0.4, 0.5, 0.6, 0.7, 0.8, 1.0, 1.2, 1.5, λ_{max} = 232 nm, λ_{max} =275 nm.

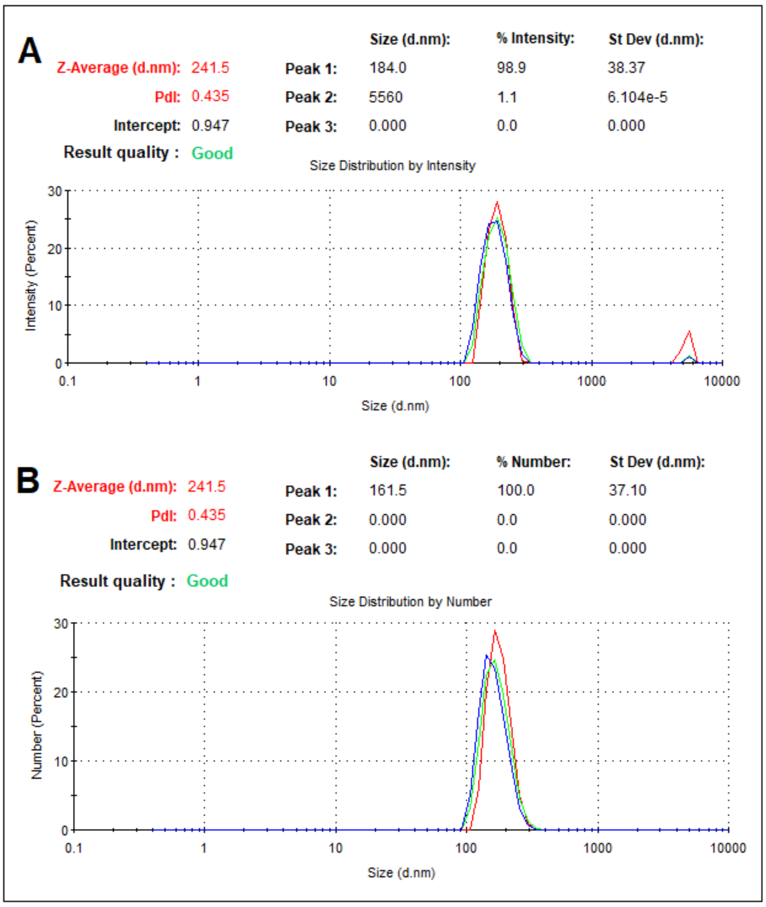

Figure S7. Particle size distribution using the Intensity (A) Number (B) parameters of BzCh in 0.1 M phosphate pH = 8.0, C_{BzCh} = 0.1 mM, 25 ºC.

3. Residual analysis

Applied to enzyme kinetics, the residual analysis is a statistical method that allows discrimination between different steady-state enzyme catalytic models. The method is based on comparison of residual sum of squares Q^2 between alternative mechanistic models:

$$Q_j^2 = \left[\sum_{i=1}^{n}\left(q_{ij}^2\right)\right]/(n - p_j)$$

with

$$q_{ij}^2 = \left(v_i - \bar{v}_{ij}\right)^2$$

n is the number of experimental points, v_i is the rate of the ith experimental point, $\bar{v}_{ij}$ is the rate determined for the ith experimental point by non-linear fitting of data according to the jth model, p_j is the number of parameters in the jth model, and q_{ij} is the ith residual in the jth model.

Data of Figs. 4A and 4B were used to calculate Q^2 for comparison of Michaelis-Menten and Webb models (n=12-16 with p =2 for Michaelis-Menten model, p=4 for Webb model). Plots of residuals *versus* fitted rates at each substrate concentration for both models were built up to 600 and 800 µM, respectively (Fig. S8-S11).

Figures show that plots of residuals *versus* predicted rates calculated by non-linear fitting exhibit an excellent correlation up to 600 µM with both the Michaelis-Menten model (Q^2= 9x10^{-7}

$\Delta A^2/min^2$) and the Webb-Radic model ($Q^2 = 5.1x10^{-7}$ $\Delta A^2/min^2$ with b=1) Thus, the Michaelis-Menten model offers the best description of the catalytic mechanism of AChE with BzCh up to 600 μM, and indicates that up to 600 μM there is no indication that binding of a second molecule of BzCh on the PAS affects the CAS. However, between 600 and 800 μM, Fig.S10 shows collapse of residuals, and Fig. S11 shows abnormally scattered data. For both models, in the BzCh concentration range from 0 to 800 μM fit do not converge and Q^2 is high (>10^{-5} $\Delta A^2/min^2$). These indicate inadequacy of both models to describe AChE-catalyzed hydrolysis of BzCh in this substrate concentration range.

Figure S8: Michaelis-Menten model

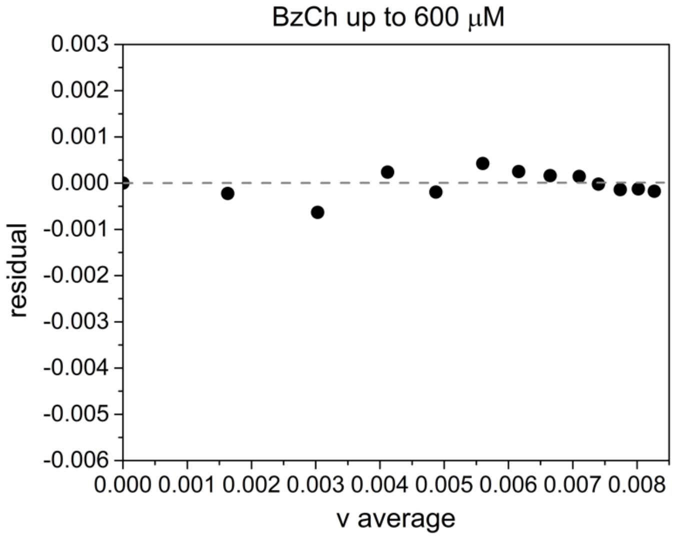

Figure S9: Webb model (Radic equation)

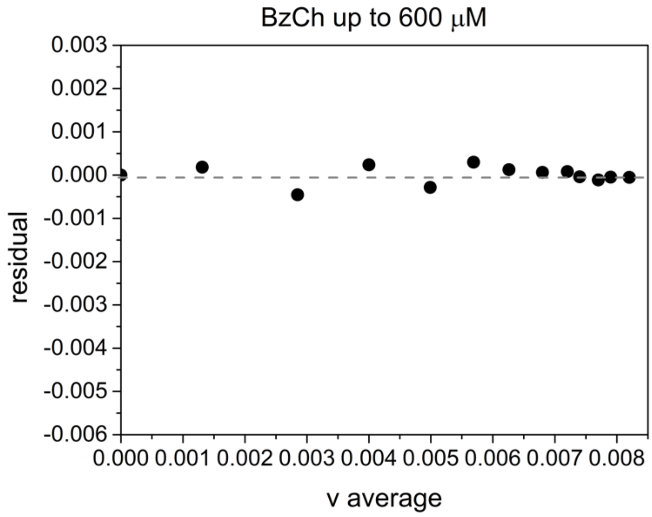

Figure S10: Michaelis-Menten model

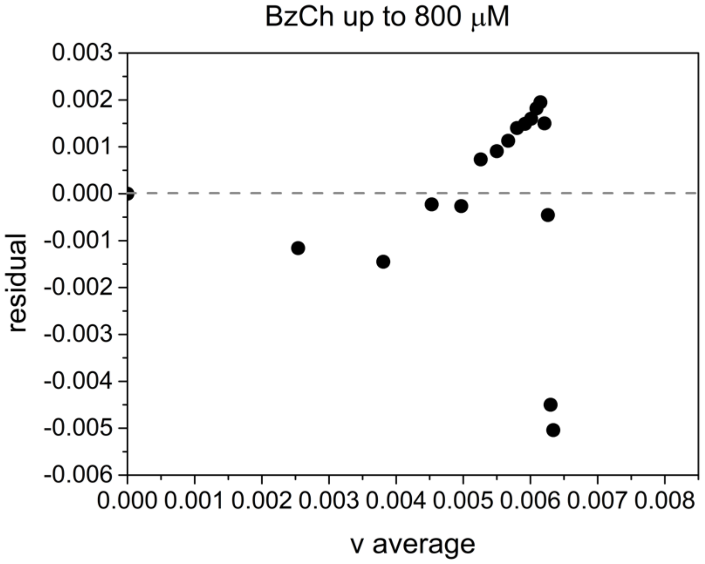

Figure S11: Webb model (Radic equation)

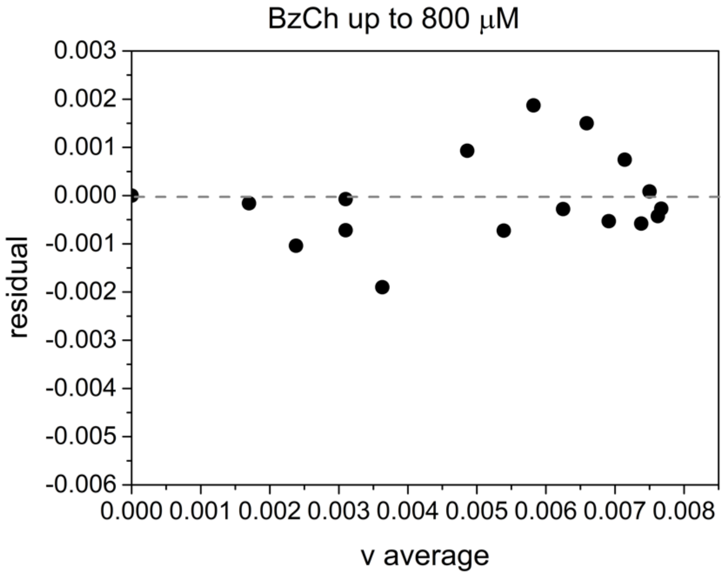

Supplement: Supplementary file 1 [file ijms-24-10472-s001.zip › ijms-2459963-supplementary.pdf]
